# Comparison of upfront haploidentical hematopoietic stem cell transplantation and salvage haploidentical hematopoietic stem cell transplantation after immunosuppressive therapy in children with acquired severe aplastic anemia - a multicenter study

**DOI:** 10.3389/fimmu.2024.1384640

**Published:** 2024-04-24

**Authors:** Danqi Luo, Yuhua Qu, Dao Wang, Benshan Zhang, Ming Sun, Hao Xiong, Jun Lu, Rui Yang, Mingyi Zhao, Haiyan Liu, Hua Jiang

**Affiliations:** ^1^ Department of Hematology, Guangzhou Women and Children’s Medical Center, Guangzhou Medical University, Guangzhou, Guangdong, China; ^2^ Department of Pediatrics, The First Affiliated Hospital of Zhengzhou University, Zhengzhou, Henan, China; ^3^ Department of Hematology, Hunan Children’s Hospital, Changsha, Hunan, China; ^4^ Department of Hematology, Wuhan Children’s Hospital, Tongji Medical College, Huazhong University of Science and Technology, Wuhan, China; ^5^ Department of Hematology, Children’s Hospital of Soochow University, Suzhou, Jiangsu, China; ^6^ Department of Pediatric Hematology, First People’s Hospital of Chenzhou, Chenzhou, Hunan, China; ^7^ Department of Pediatrics, The Third Xiangya Hospital, Central South University, Changsha, Hunan, China

**Keywords:** upfront Haplo-HSCT, salvage Haplo-HSCT, IST, SAA, children, multicenter study

## Abstract

**Background:**

For children with severe aplastic anemia, if the first immunosuppressive therapy (IST) fails, it is not recommended to choose a second IST. Therefore, for patients without matched sibling donor (MSD) and matched unrelated donor (MUD), haploidentical hematopoietic stem cell transplantation (Haplo-HSCT) can be chosen as a salvage treatment. This article aims to explore the comparison between upfront Haplo-HSCT and salvage Haplo-HSCT after IST.

**Methods:**

29 patients received salvage Haplo-HSCT, and 50 patients received upfront Haplo-HSCT. The two groups received Bu (Busulfan, 3.2mg/kg/d*2d on days -9 to-8), CY (Cyclophosphamide, 60mg/kg/d*2d on days -4 to-3), Flu (fludarabine, 40mg/m^2^/d*5d on days -9 to -5) and rabbit ATG (Anti-thymocyte globulin, total dose 10mg/kg divided into days -4 to -2).

**Results:**

The OS of the salvage Haplo-HSCT group showed no difference to the upfront Haplo-HSCT group (80.2 ± 8.0% vs. 88.7 ± 4.8%, p=0.37). The FFS of the salvage Haplo-HSCT group also showed no difference to the frontline Haplo-HSCT group (75 ± 8.2% vs. 84.9 ± 5.3%, p=0.27). There was no significant difference in the incidence of other complications after transplantation between the two groups, except for thrombotic microangiopathy (TMA). In the grouping analysis by graft source, the incidence of II-IV aGVHD in patients using PBSC ± BM+UCB was lower than that in the PBSC ± BM group (p=0.010)

**Conclusion:**

Upfront Haplo-HSCT and salvage Haplo-HSCT after IST in children with acquired severe aplastic anemia have similar survival outcomes. However, the risk of TMA increases after salvage Haplo-HSCT. This article provides some reference value for the treatment selection of patients. In addition, co-transplantation of umbilical cord blood may reduce the incidence of GVHD.

## 1 Introduction

The treatment of severe aplastic anemia (SAA) in children includes immunosuppressive therapy (IST) and hematopoietic stem cell transplantation (HSCT). IST mainly relies on anti-thymocyte globulin (ATG) and cyclosporine (CsA) as skeletons. According to the consensus in China, matched sibling donor (MSD) HSCT and matched unrelated donor (MUD) HSCT are recommended for patients under 50 years old. If the patient does not have MSD or MUD, haploidentical hematopoietic stem cell transplantation (Haplo-HSCT) can also be considered in well-experienced transplant centers (1). Similarly, the Japanese guidelines stratified the risk factors for SAA in children. In emergencies, patients with short telomere or PNH clones can choose Haplo-HSCT instead of IST (2). For pediatric patients who choose IST on the first line, there may be three outcomes: the first being responsive to IST without recurrence, the second being responsive to IST treatment then relapsed, and the third being consistently unresponsive to IST, defined as refractory. The response rates of retreatment with horse-ATG or rabbit ATG (r-ATG) in refractory or relapsed patients have varied significantly, from 22% to 77%. However, the average age of the subjects in these studies is mostly greater than 18 years old, and they are not solely targeted at pediatric patients (3–8). For children, Seiji Kojima et al.’s prospective study suggested that salvage alternative donor HSCT therapy is superior to repeated IST, and that pediatric patients are more susceptible to the currently used enhanced ATG therapy than adult patients, and a single ATG process is sufficient to identify their response to these immunosuppressive agents. In addition, IST increases the risk of clone evolution cannot be ignored. Overall, for children, if they fail to respond to an initial IST, it is not recommended to choose a second IST treatment (9).

When selecting the types of grafts for transplantation, G-CSF mobilized PBSC (G-PB) is a faster and more practical alternative to BM as a source of stem cells. However, G-CSF mobilized BM can also result in faster implantation of neutrophils and platelets. Furthermore, compared to PBSC, BM appears to result in fewer cases of acute and chronic GVHD (10). Recently, Haplo-HSCT combined with a third-party umbilical cord blood (UCB) unit has been used in clinical trials. UCB has fewer mature or primed T cells that are predominant in inducing GVHD compared with peripheral blood grafts. Additionally, UCB is a rich and rapidly accessible source of several immunomodulatory cells, such as regulatory T cells (Tregs) and mesenchymal stromal cells (MSCs), both of which have been found to be effective in controlling severe GVHD (11). However, because the number of stem cells derived from the donor is one order of magnitude larger than that of third-party cord blood, the cord blood will be rejected under normal circumstances, ultimately achieving donor chimerism.

Therefore, for patients without MSD and MUD, Haplo-HSCT can be a relatively good choice. We conducted a retrospective multicenter study to compare upfront Haplo-HSCT and salvage Haplo-HSCT after IST.

## 2 Methods

### 2.1 Patients

From December 12, 2015, to September 26, 2022, A total of 79 patients were included in our retrospective multicenter study, with data from 6 hospitals, namely First People’s Hospital of Chenzhou, Children’s Hospital of Soochow University, Wuhan Children’s Hospital, The First Affiliated Hospital of Zhengzhou University, Hunan Children’s Hospital, Guangzhou Women’s and Children’s Medical Center. All patients were diagnosed according to standard diagnostic methods (12) and excluded from MDS, inherited bone marrow failure syndrome, and all patients who underwent testing had negative PNH clones. Patients with any severe liver, heart, lung or kidney disease, or any active infection were excluded. Receiving upfront Haplo-HSCT is defined as not using IST before transplantation or only using CsA for less than three months (<3 months). Salvage Haplo-HSCT after IST is defined as using CsA and ATG or using CsA for more than three months (≥3 months) before transplantation. Twenty-nine patients received salvage Haplo-HSCT, and 50 patients received upfront Haplo-HSCT. The average age of the study subjects is 7.07 ± 3.07 years. The time from diagnosis to transplantation is 2.9 ± 7.5months in the upfront Haplo-HSCT group and 14.6 ± 19.3 months in the salvage Haplo-HSCT group. The median follow-up time is 30.46 ± 22.49 months. Patient characteristics are shown in **Table 1**.

**Table 1:** Patient characteristics.

		Upfront Haplo-HSCT (N=50)	Salvage Haplo-HSCT (N=29)	*p*
Age(years)		6.8 ± 2.8	7.5 ± 3.5	0.326
Gender	Female	21 (42%)	15 (51.7%)	0.547
	Male	29 (58%)	14 (48.3%)	
Time to HSCT (months)		2.9 ± 7.5	14.6 ± 19.3	0.004
Donor. Gender	Female	15 (30%)	13 (44.8%)	0.278
	Male	35 (70%)	16 (55.2%)	
Donor. Age		28.2 ± 10.1	27.1 ± 11.2	0.655
ABO match	Matched	31 (62%)	14 (48.3%)	0.002
	Minor mismatched	12 (24%)	4 (13.8%)	
	Major mismatched	3 (6%)	11 (37.9%)	
	Different	4 (8%)	0 (0%)	
Graft.Type	PBSC ± BM	14 (28%)	3 (10.3%)	0.120
	PBSC ± BM+UCB	36 (72%)	26 (89.7%)	
Ferritin	<1000ng/L	39 (78%)	17 (58.6%)	0.116
	>1000ng/L	11 (22%)	12 (41.4%)	
Neutrophil engraftment(days)		13.6 ± 2.4	13.7 ± 3.0	0.792
Platelet engraftment(days)		16.5 ± 6.1	18.6 ± 9.7	0.303

### 2.2 Graft types

**Figure 1:**
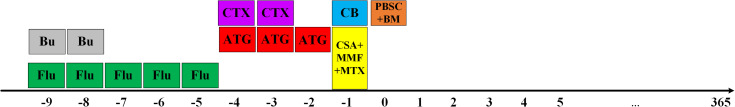
Schematic diagram of conditioning regimen.

The selection of graft types includes PBSC ± BM or PBSC ± BM+UCB. To mobilize stem cells, rhG-CSF were subcutaneously injected into donors on days −4 to 0 (5 μg/kg/day). The target CD34+ should reach 2.5(10^6/kg), and the number of mononuclear cells was less than 10 (10^8/kg). The umbilical cord blood (UCB) units were obtained from the cord blood bank across China. The choice of UCB was according to HLA typing (from 6/6 to 4/6), cell count (from high to low), blood type (from matched to mismatched) and the patient’s preference. Days before the first and last stem cell infusion were designated by a minus (–) sign and a plus (+) sign, respectively. If choosing to use UCB in combination, a single UCB fusion was conducted on day-1 (**Figure 1**).

### 2.3 Conditioning regimen

The two groups received Bu (Busulfan, 3.2mg/kg/d*2d on days -9 to-8), CY (Cyclophosphamide, 60mg/kg/d*2d on days -4 to-3), Flu (fludarabine, 40mg/m^2^/d*5d on days -9 to -5) and rabbit ATG (Anti-thymocyte globulin, total dose 10mg/kg divided into days -4 to -2) (**Figure 1**).

### 2.4 GVHD prophylaxis

For prophylaxis of GVHD, CsA is routinely used intravenously from day-1, with the concentration maintained at 200-250ng/ml, and the dosage will be reduced after six months of use, and it will be stopped for about one year. In the case of liver GVHD, adjust to FK506 and keep the concentration between 8-12ng/ml. Mycophenolate mofetil (MMF) is routinely used, 30mg/kg per day, divided into twice or three times, until 30 days after transplantation. Methotrexate (MTX) is administered at a dosage of 15mg/m2 on day+1, 10mg/m2 on day+3, and day+6, respectively. G-CSF was used to increase the neutrophil count on day+5.

### 2.5 Definition of engraftment and evaluation of outcomes

*Neutrophil engraftment* is defined as ANC ≥0.5×10^9/L for three consecutive days, *platelet engraftment* is defined as platelets ≥20×10^9/L without transfusion for seven consecutive days, and red blood cell engraftment is defined as hemoglobin not less than 80g/L without transfusion. *Graft failure* is defined as the failure to achieve hematopoietic recovery after transplantation, with all or part of the hematopoietic cells derived from the recipient. Chimerism was detected by using fluorescence *in situ* hybridization probes to detect peripheral blood for sex-mismatched pairs or short tandem repeats of polymorphic DNA sequences for sex-matched pairs. After chimerism detection, only donor-type hematopoietic cells after allogeneic HSCT are defined as complete donor chimerism (13). To evaluate outcomes, three-year overall survival (OS) and failure-free survival (FFS) were estimated. The OS is calculated from the transplant date to the final follow-up date. The definition of FFS is the survival rate without treatment failure. Death, graft rejection, recurrence and the need for remedial treatment are considered as treatment failures (14).

### 2.6 Statistical methods

Continuous variables are expressed as mean ± standard deviation (SD) or median and interquartile spacing, while categorical data are expressed as quantity (N) and percentage (%). Kruskal-Wallis test and analysis of variance (ANOVA)were used for continuous variables, and the Chi-square test or Fisher’s exact test were used for categorical variables. In addition, a subgroup analysis of graft types was also conducted. The relationship between independent variables and OS and FFS was analyzed using Cox regression. All independent variables are included in univariate analysis, and significant variables in univariate analysis are included in multivariate analysis. The probability of OS and FFS and survival curves are obtained by the Kaplan – Meier method. Log-rank test was used to compare the survival rate among groups. Compare the number of platelets, neutrophils, and lymphocytes in the first, third, and sixth month and donor chimerism rate at 1-month post-transplantation between the two groups to evaluate hematopoietic reconstitution. All statistical analyses were conducted using IBM SPSS version 25 and R project (4.2.2). A P-value of <0.05 was considered statistically significant.

## 3 Results

### 3.1 Patient characteristics

**Table 1** shows the clinical characteristics of 79 patients. There were no significant differences between the two groups in terms of age, gender, donor age, gender, graft type, serum ferritin levels, average time of neutrophil and platelet implantation. The salvage Haplo-HSCT group was longer than the upfront Haplo-HSCT group in terms of time to diagnosis to transplantation before transplantation(14.6 ± 19.3 months VS 2.9 ± 7.5 months, p=0,004), and there are differences in the distribution of ABO matching between the two groups (p=0.002).

### 3.2 Complications after transplantation

Only thrombotic microangiopathy (TMA) showed differences between the two groups, and the salvage Haplo-HSCT group had a higher probability of developing TMA (0% VS 13.8%, p=0.031, **Table 2**). The four patients who experienced TMA were all in the salvage Haplo-HCT group. Patient 1 developed TMA more than three months after transplantation, underwent plasma exchange, discontinued IST, and treatment with rituximab and basiliximab, but was unable to control the condition. Eventually, patient 1 died due to cytomegalovirus encephalitis, GVHD, and TMA. Patient 2 was cured under the treatment of rituximab, heparin, steroid hormones, and ruxolitinib. Patient 3 developed TMA on day +17 after transplantation and was cured after discontinuing cyclosporine. Patient 4 developed TMA at two months and eight months after transplantation and was treated with plasma exchange, rituximab, corticosteroids, basiliximab, and infliximab. Patient 4 died due to TMA, IV GVHD, pulmonary infection (Aspergillus), and cytomegalovirus infection one year after transplantation. There was no significant difference in other complications between the two groups, such as aGVHD, cGVHD, Posterior reversible encephalopathy syndrome, Veno-occlusive disease, Cytomegalovirus infection, Epstein-Barr virus infection, Post-transplant Lymphoproliferative Disorder, Graft failure and Poor graft function. In the upfront Haplo-HSCT group, 12 patients (24%) developed grade III-IV acute graft versus host disease (aGVHD), and 3 patients (6.0%) developed intensive chronic graft versus host disease(cGVHD). In the salvage Haplo-HSCT group, the incidence of III-IV aGVHD and intensive cGVHD was 10.3% and 3.4%, respectively (**Table 2**). In the grouping analysis by graft source, the incidence of II-IV aGVHD in patients using PBSC ± BM+UCB was lower than that in the PBSC ± BM group (32.3% vs 70.6%, p=0.010, **Table 3**). In subgroup analysis of graft types (**Table 4**), for the upfront Haplo-HSCT group, the incidence of II-IV aGVHD in patients using PBSC ± BM+UCB as the graft source was lower than that in the PBSC ± BM group (30.6% vs 78.6%, p=0.006). For the salvage Haplo-HSCT group, using PBSC ± BM+UCB as the graft source can reduce the severity of cGVHD (p=0.009).

**Table 2:** Complications after Transplantation.

		Upfront Haplo-HSCT (N=50)	Salvage Haplo-HSCT (N=29)	*p*
aGVHD				
	II-IV	22 (44%)	10 (34.5%)	0.553
	III-IV	12 (24%)	3 (10.3%)	0.232
cGVHD	No	35 (70%)	21 (72.4%)	0.882
	Limited	12 (24%)	7 (24.1%)	
	Extensive	3 (6%)	1 (3.4%)	
Hemorrhagic cystitis,yes		11 (22%)	6 (20.7%)	1.000
PRES,yes		4 (8%)	3 (10.3%)	1.000
VOD,yes		3 (6%)	2 (6.9%)	1.000
CMV,yes		32 (64%)	20 (69%)	0.840
EBV,yes		22 (44%)	11 (37.9%)	0.771
TMA,yes		0 (0%)	4 (13.8%)	0.031*
PTLD,yes		2 (4%)	1 (3.4%)	1.000
GF,yes		2 (4%)	3 (10.3%)	0.524
PGF,yes		1 (2%)	1 (3.4%)	1.000

* p<0.05, aGVHD, acute graft versus host disease; cGVHD, chronic graft versus host disease; PRES, Posterior reversible encephalopathy syndrome; VOD, Veno-occlusive disease; CMV, Cytomegalovirus; EBV, Epstein-Barr virus; TMA, Thrombotic microangiopathy; PTLD, Post-transplant Lymphoproliferative Disorder; GF, graft failure; PGF, poor graft function.

**Table 3:** The impact of graft types on implantation time and complications.

		PBSC ± BM (N=17)	PBSC ± BM+UCB (N=62)	*p*
Neutrophil engraftment(days)		12.3 ± 2.1	14.0 ± 2.7	0.019
Platelet engraftment(days)		14.7 ± 4.3	18.0 ± 8.2	0.028
aGVHD				
	II-IV	12 (70.6%)	20 (32.3%)	0.010
	III-IV	5 (29.4%)	10 (16.1%)	0.375
cGVHD	No	10 (58.8%)	46 (74.2%)	0.269
	Limited	5 (29.4%)	14 (22.6%)	
	Extensive	2 (11.8%)	2 (3.2%)	
Hemorrhagic cystitis,yes		7 (41.2%)	10 (16.1%)	0.058
PRES,yes		1 (5.9%)	6 (9.7%)	0.995
VOD,yes		1 (5.9%)	4 (6.5%)	1.000
CMV,yes		13 (76.5%)	39 (62.9%)	0.450
EBV,yes		8 (47.1%)	25 (40.3%)	0.825
TMA,yes		0 (0%)	4 (6.5%)	0.652
PTLD,yes		0 (0%)	3 (4.8%)	0.835
GF,yes		0 (0%)	5 (8.1%)	0.517
PGF,yes		0 (0%)	2 (3.2%)	1.000
Overall survival		16 (94.1%)	53 (85.5%)	0.591

aGVHD, acute graft versus host disease; cGVHD, chronic graft versus host disease; PRES, Posterior reversible encephalopathy syndrome; VOD, Veno-occlusive disease; CMV, Cytomegalovirus; EBV, Epstein-Barr virus; TMA, Thrombotic microangiopathy; PTLD, Post-transplant Lymphoproliferative Disorder; GF, graft failure; PGF, poor graft function.

**Table 4:** Subgroup analysis of graft types.

		upfront Haplo-HSCT			salvage Haplo-HSCT		
		PBSC ± BM (N=14)	PBSC ± BM+UCB (N=36)	*p*	PBSC ± BM (N=3)	PBSC ± BM+UCB (N=26)	*p*
II-IVaGVHD,yes		11 (78.6%)	11 (30.6%)	0.006	1 (33.3%)	9 (34.6%)	1.000
III-IVaGVHD,yes		5 (35.7%)	7 (19.4%)	0.400	0 (0%)	3 (11.5%)	1.000
cGVHD	No	8 (57.1%)	27 (75%)	0.446	2 (66.7%)	19 (73.1%)	0.009
	Limited	5 (35.7%)	7 (19.4%)		0 (0%)	7 (26.9%)	
	Extensive	1 (7.1%)	2 (5.6%)		1 (33.3%)	0 (0%)	
Hemorrhagic cystitis,yes		5 (35.7%)	6 (16.7%)	0.280	2 (66.7%)	4 (15.4%)	0.186
PRES,yes		1 (7.1%)	3 (8.3%)	1.000	0 (0%)	3 (11.5%)	1.000
VOD,yes		1 (7.1%)	2 (5.6%)	1.000	0 (0%)	2 (7.7%)	1.000
CMV,yes		10 (71.4%)	22 (61.1%)	0.723	3 (100%)	17 (65.4%)	0.570
EBV,yes		8 (57.1%)	14 (38.9%)	0.395	0 (0%)	11 (42.3%)	0.423
TMA,yes		0	0		0 (0%)	4 (15.4%)	1.000
PTLD,yes		0 (0%)	2 (5.6%)	0.923	0 (0%)	1 (3.8%)	1.000
GF,yes		0 (0%)	2 (5.6%)	0.923	0 (0%)	3 (11.5%)	1.000
PGF,yes		0 (0%)	1 (2.8%)	1.000	0 (0%)	1 (3.8%)	1.000

aGVHD, acute graft versus host disease; cGVHD, chronic graft versus host disease; PRES, Posterior reversible encephalopathy syndrome; VOD, Veno-occlusive disease; CMV, Cytomegalovirus; EBV, Epstein-Barr virus; TMA, Thrombotic microangiopathy; PTLD, Post-transplant Lymphoproliferative Disorder; GF, graft failure; PGF, poor graft function.

### 3.3 Engraftment and hematopoietic reconstruction

There was no significant difference in the average time of neutrophil and platelet implantation between the two groups (p>0.05, **Table 1**). Two patients (4%) in the upfront Haplo-HSCT group experienced graft failure, while three patients (10.3%) in the salvage Haplo-HSCT group experienced graft failure (p=0.524, **Table 2**). **Table 5** reflects the hematopoietic reconstruction situation of the two groups, and overall, the speed of hematopoietic reconstruction in the salvage Haplo-HSCT group is equal to that in the upfront Haplo-HSCT group.

**Table 5:** Hematopoietic reconstruction.

	upfront Haplo-HSCT	salvage Haplo-HSCT	*p*
Neutrophils(10^9/L)			
1st-month	4.1 ± 3.6	3.1 ± 2.5	0.189
3rd-month	2.4 ± 1.7	4.5 ± 12.6	0.396
6th-month	2.6 ± 1.6	2.6 ± 1.9	0.901
			
Lymphocytes(10^9/L)			
1st-month	0.9 ± 0.8	0.7 ± 0.7	0.146
3rd-month	1.4 ± 1.0	2.3 ± 4.5	0.31
6th-month	1.8 ± 1.0	1.9 ± 1.5	0.724
Platelets(10^9/L)			
1st-month	140.4 ± 87.2	112.7 ± 65.5	0.142
3rd-month	187.2 ± 111.1	156.6 ± 88.8	0.217
6th-month	211.8 ± 97.1	188.7 ± 123.1	0.389
Donor chimerism rate(%, +30d)			
	97.19 ± 13.51	99.48 ± 1.58	0.242

### 3.4 Survival

**Figure 2:**
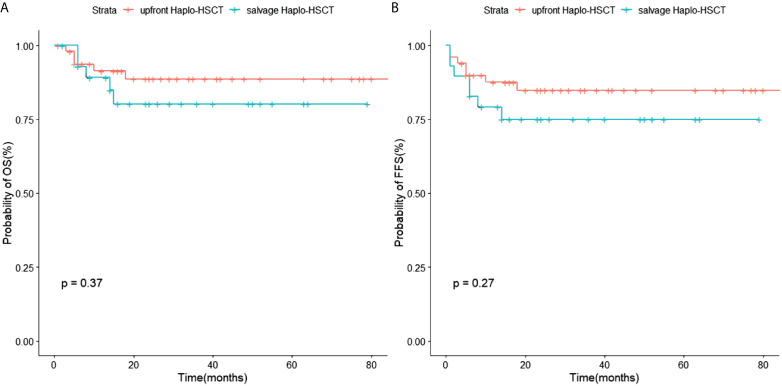
Kaplan-Meier estimates of **(A)** OS of upfront Haplo-HSCT and salvage Haplo-HSCT; **(B)** FFS of upfront Haplo-HSCT and salvage Haplo-HSCT.

The OS of the salvage Haplo-HSCT group showed no difference to the frontline Haplo-HSCT group (80.2 ± 8.0% vs. 88.7 ± 4.8%, p=0.37) (**Figure 2A**). The FFS of the salvage Haplo-HSCT group also showed no difference to the frontline Haplo-HSCT group (75 ± 8.2% vs 84.9 ± 5.3%, p=0.27) (**Figure 2B**). Of the 79 patients, a total of 10 died due to infection (n=3), multiple organ failure (n=3), hemorrhagic shock (n=2), cerebral hemorrhage (n=1), and TMA(n=1). In univariate and multivariate Cox regression, only statistically significant independent variables were listed, and the results showed that the occurrence of Post-transplant Lymphoproliferative Disorder (PTLD) is a risk factor for FFS. For the upfront Haplo-HSCT group, prolonged platelet implantation time is a risk factor for OS and FFS (**Table 6**).

**Table 6:** Results of Cox regression.

	Univariate analysis		Multivariate analysis	
	HR [95%CI]	*p*	HR [95%CI]	*p*
OS				
TMA	5.64(1.179-26.97)	0.030*	1.35(0.44-33.73)	0.221
FFS				
III-IV GVHD	2.961(0.987-8.883)	0.052	2.877(0.944-8.764)	0.062
PTLD	8.565(1.894-38.74)	0.005**	8.243(1.753-38.740)	0.007**
OS in upfront Haplo-HSCT				
Platelet engraftment(days)	1.147(1.042-1.263)	0.005**	1.131(1.027-1.246)	0.011*
FFS in upfront Haplo-HSCT				
Platelet engraftment(days)	1.133(1.047-1.226)	0.001**	1.123(1.032-1.221)	0.007**
OS in salvage Haplo-HSCT	None		None	
FFS in salvage Haplo-HSCT				
III-IV GVHD	5.395(0.985-29.54)	0.052	2.956(0.330-26.47)	0.333
PTLD	39.24(2.359-652.7)	0.010*	15.148(0.542-422.89)	0.110

*p<0.05, ** p<0.01, aGVHD, acute graft versus host disease; TMA, Thrombotic microangiopathy; PTLD, Post-transplant Lymphoproliferative Disorder.

## 4 Discussion

Previous studies have shown that the OS of first-line Haplo-HSCT is comparable to IST, but the FFS and health-related quality of life of Haplo-HSCT are superior to IST treatment (15). However, due to the high risk of transplantation, patients may prefer to prioritize IST treatment. For patients who have failed IST treatment and do not have a matching donor, salvage Haplo-HSCT can be chosen. However, there are currently few studies that directly compare the survival outcomes of upfront Haplo-HSCT and salvage Haplo-HSCT. Therefore, our study aims to compare upfront Haplo-HSCT and salvage Haplo-HSCT post-IST, providing a basis for the selection of treatment for children with SAA. In our study, the baseline features of the two groups of patients were almost matched, making the comparison of results between the two groups more reliable.

In terms of complications after transplantation, there was only a difference in the incidence of TMA between the two groups, and the salvage Haplo-HSCT group had a higher incidence of TMA, which may be related to the longer use of cyclosporine before transplantation. Previous studies have shown that cyclosporine may damage vascular endothelium, and exposure to prior calcineurin inhibitors is a risk factor for transplant-associated TMA (16, 17). In the past few years, the probability of developing III - IV aGVHD in patients with Haplo-HSCT based on G-CSF and ATG protocols was 4.9-29.6% (18), the incidence of aGVHD in our groups remains relatively low. Our study showed no significant difference in the frequency of aGVHD and cGVHD between the two groups, which is like the study by Zhu et al., who also mainly used Haplo-HSCT for salvage treatment. They concluded that the incidence of II-IV aGVHD for frontline Haplo-HSCT and salvage Haplo-HSCT was comparable (19). However, another study targeting pediatric patients, with matched family donor (MFD) as the main source of salvage treatment, found that the cumulative incidence of II-IV aGVHD of HSCT post IST failure was higher than MFD HSCT (25.0 ± 5.0% vs 8.0 ± 1.0%, P<0.0001). The five-year cumulative incidence of cGVHD was also higher in HSCT post-IST compared to MFD (20.0 ± 4.0% vs 6.0± 2.0%, P<0.0001) (20). Another study focuses on teenagers, cumulative incidence of aGVHD was similar in MFD transplants and transplants post failed IST(P=0.18). cGVHD was higher in transplants post failed IST than in MFD HSCT (P=0.0009) (21). In addition, our research findings suggest that co-transplantation with cord blood may reduce the incidence of GVHD, which is consistent with previous research findings. A study by Wu et al. included 91 children (average age 1-16 years old) based on the “Beijing protocol”, and the conclusion was that the incidence of GVHD in the haplo-cord group is lower compared to the haplo group (11).

In our study, hematopoietic reconstitution in the salvage Haplo-HSCT group was almost equal to the front-line Haplo-HSCT group. In multivariate analysis, the occurrence of PTLD is an adverse factor for FFS, but there is no difference in the frequency of occurrence between the two groups. Therefore, based on the above situation, there is no statistically significant difference in OS and FFS between the two groups. Zhu et al.’s study showed that the EFS was lower in the salvage HSCT group compared with the Haplo-HSCT group (41.7 ± 14.2% versus 80.0 ± 8.9%; P=0.046) (19) Marsh, Judith et al.’s research suggested that OS was comparable between 86% in the MFD HSCT group, 90% in patients given front-line IST alone, and 78% in transplantation post failed front-line IST (P=0.14). EFS in the same groups was respectively 83%, 64% and 71% (P=0.04) (21). Wu D et al.’s research showed that the estimated OS and FFS of salvage Haplo-HSCT at three years was 76.30 ± 9.70%, and the median age of research subjects was 26 years (10–54 years) (22). Yoo, K H et al.’s research suggested that the estimated EFS of the frontline HSCT group was higher than that of the salvage HSCT group (91.3% vs 50.9%, P = 0.015) (23). On the contrary, Xu et al. believed that there was no significant difference in II-IV aGVHD (P=0.699), cGVHD (P=0.916), OS (P=0.698), and FFS (P=0.899) between the two groups for children receiving upfront Haplo-HSCT or salvage Haplo-HSCT (24). Therefore, we believe that for experienced transplant centers, patients with generally acceptable conditions, and voluntarily, patients without MSD and MUD can actively receive upfront Haplo-HSCT. Of course, salvage Haplo-HSCT after IST failure is advisable, but it carries a higher risk of TMA. In addition, for well-experienced transplant centers, choosing upfront Haplo-HSCT can reduce the drug side effects of IST and avoid the risk of anxiety for parents and worsening infection when IST is ineffective for a long time.

However, our research has some limitations. Firstly, the sample size of the salvage treatment group is small. Secondly, as this study is retrospective, there may be other possible factors that have yet to be included in the analysis. In future research, we need a well-designed prospective study to verify our findings and increase our sample size.

## 5 Conclusion

Upfront Haplo-HSCT and salvage Haplo-HSCT after IST in children with acquired severe aplastic anemia have similar survival outcomes. However, the risk of TMA increases after salvage Haplo-HSCT. This article provides some reference value for the treatment selection of patients. In addition, co-transplantation of umbilical cord blood may reduce the incidence of GVHD.

## Data availability statement

The original contributions presented in the study are included in the article/supplementary material. Further inquiries can be directed to the corresponding author.

## Ethics statement

The studies involving humans were approved by Guangzhou Women and Children’s Medical Research Ethics Committee, and the committee’s reference number is 27601[2019]. The studies were conducted in accordance with the local legislation and institutional requirements. Written informed consent for participation in this study was provided by the participants’ legal guardians/next of kin.

## Author contributions

DL: Formal analysis, Writing – original draft. YQ: Data curation, Writing – original draft. DW: Data curation, Writing – review & editing. BZ: Data curation, Writing – review & editing. MS: Data curation, Writing – review & editing. HX: Data curation, Writing – review & editing. JL: Data curation, Writing – review & editing. RY: Data curation, Writing – review & editing. MZ: Methodology, Supervision, Writing – review & editing. HL: Funding acquisition, Methodology, Writing – original draft. HJ: Formal analysis, Funding acquisition, Methodology, Supervision, Validation, Visualization, Writing – review & editing.
